# Practical Illustration of the Concours in France

**Published:** 1850-11

**Authors:** 


*Practical Illustration of the Concours in France.*—Dr. Warren, in his published Address, gives the following account of the mode in which Dupuytren achieved success at the *Concours* for the place of Chief Surgeon of the Hotel Dieu:—"In the evening after the *Concours*, Dupuytren visited the house of a friend of his, of high political importance, and, rushing into his room, struck his hands upon his head, exclaiming, "I am lost, Roux will triumph!" His friend endeavored to pacify him; inquired into the circumstances which had caused his discouragement, and discovered that his depression was produced by the apprehension that the supporters of M. Roux were more powerful than his own. His friend then told him, "Obey me, and you shall still be victorious. Go this moment to Madame B.; her influence is all-powerful. She has a good opinion of you, and will be flattered by your application, and gratified at mixing in a court intrigue. Throw yourself at her feet, supplicate her to exercise her power in your favor, and never leave her till she has given her promise to aid you." Dupuytren obeyed. He visited the lady, obtained her promise, and was subsequently declared the successful candidate. This occurrence is a practical commentary on the infallible impartiality said to characterize the mode of election by *Concours*." The *Concours* has been proposed as the mode of electing Professors in the Medical Institutions of this country: could a more striking instance of favoritism be found, under our present system, than that by which the most distinguished of modern surgeons obtained his appointment!

